# Supplementary material for: Effect of Ipomoeassin F on the Synthesis of Membrane and Secretory Proteins in Triple-Negative Breast Cancer Cells
Source: ACS Omega. 2025 Aug 19;10(34):38522–30. doi: 10.1021/acsomega.5c02784 (PMC12409539; doi:10.1021/acsomega.5c02784)
Supplement: Supplementary file 1 [file ao5c02784_si_001.pdf]

Supporting Information

Effect of ipomoeassin F on the synthesis of membrane and secretory proteins in triple-negative breast cancer cells

Brihget Sicairos[1], Jianhong Zhou[1], Zhijian Hu[2], Qingyang Zhang[3,*], Wei Q Shi[4,*], and Yuchun Du[1,*]

[1] Department of Biological Sciences, University of Arkansas, Fayetteville, Arkansas 72701, USA.

[2] Feinstein Institute for Medical Research, Northwell Health, 350 Community Dr., Manhasset, New York, 11030, USA.

[3] Department of Mathematical Sciences, University of Arkansas, Fayetteville, Arkansas 72701, USA.

[4] Department of Chemistry, Ball State University, Muncie, Indiana 47306, USA.

*Corresponding authors: qz008@uark.edu; wqshi@bsu.edu; ydu@uark.edu

Contents

1. Figure S1…………………………………………………………………………S2
2. Table S1–S6 ………………………………………………….See separate Excel file

Figure S1

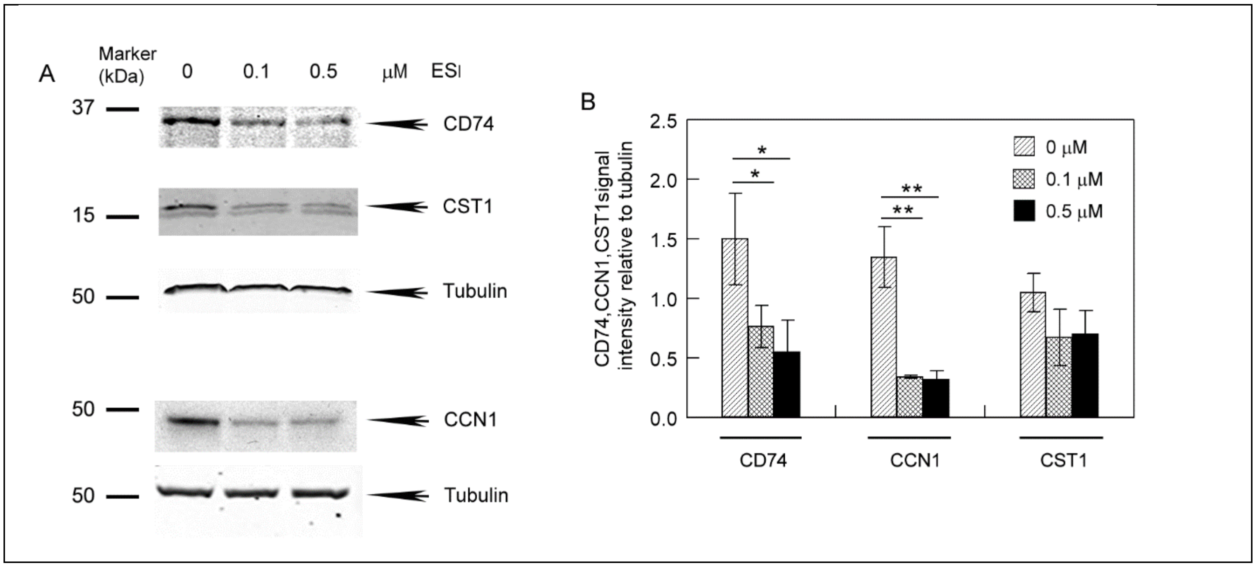

Figure S1. Effect of eeyarestatin I (ES_I), a potent Sec61 inhibitor, on the expression of the Ipom-F-sensitive proteins CD74, CCN1, and CST1 in MDA-MB-231 cells. Cells were mock-treated (0 nM) or treated with 0.1 μM or 0.5 μM ES_I for 12 hours, and the expression levels of the indicated proteins were determined by Western blotting. (A) Representative Western blot results for each protein. (B) Quantification of Western blot signal intensities for CD74, CCN1, and CST1. $^*p < 0.05$, $^{**}p < 0.01$. As shown, the expression of CD74 and CCN1 were significantly inhibited by ES_I. The expression of CST1 was not significantly inhibited by ES_I, but the p-values approached the threshold for statistical significance (p = 0.084 and 0.077 at 0.1 μM and 0.5 μM, respectively, compared to 0 μM). All experiments were performed in three independent biological replicates. The molecular weight marker used for Western blotting was the Precision Plus Protein Dual Color Standards from Bio-Rad (Catalog # 1610374).
